# Supplementary material for: Hot spot prediction in protein-protein interactions by an ensemble system
Source: BMC Syst Biol. 2018 Dec 31;12(Suppl 9):132. doi: 10.1186/s12918-018-0665-8 (PMC6311905; doi:10.1186/s12918-018-0665-8)
Supplement: Supplementary file 2 — All relevant MATLAB code. A simple Matlab implement of our predictor. (ZIP 19,742 kb) [file 12918_2018_665_MOESM2_ESM.zip › Additional file 2/Read ASA/ASA website/dbMPIKT/3W2DA.html]

 NetSurfP - Protein Surface Accessibility and Secondary Structure Predictions

  

|  |  |  |
| --- | --- | --- |
|  |  | NetSurfP - Protein Surface Accessibility and Secondary Structure Predictions  Technical University of Denmark |

  


---

```
# For publication of results, please cite:
# A generic method for assignment of reliability scores applied to solvent accessibility predictions.
# Bent Petersen, Thomas Nordahl Petersen, Pernille Andersen, Morten Nielsen and Claus Lundegaard
# BMC Structural Biology 2009, 9:51 doi:10.1186/1472-6807-9-51
#
# Column 1: Class assignment - B for buried or E for Exposed - Threshold: 25% exposure, but not based on RSA
# Column 2: Amino acid
# Column 3: Sequence name
# Column 4: Amino acid number
# Column 5: Relative Surface Accessibility - RSA
# Column 6: Absolute Surface Accessibility
# Column 7: Z-fit score for RSA prediction
# Column 8: Probability for Alpha-Helix
# Column 9: Probability for Beta-strand
# Column 10: Probability for Coil
E E  3W2DA                  1    0.924 161.353   0.601   0.003   0.003   0.994
E S  3W2DA                  2    0.697  81.665  -0.628   0.058   0.017   0.925
E Q  3W2DA                  3    0.588 104.963  -0.158   0.058   0.017   0.925
E P  3W2DA                  4    0.627  89.042  -0.733   0.058   0.017   0.925
E D  3W2DA                  5    0.660  95.120  -0.104   0.018   0.019   0.964
B P  3W2DA                  6    0.194  27.472  -1.127   0.018   0.019   0.964
E K  3W2DA                  7    0.517 106.388   1.486   0.016   0.005   0.979
E P  3W2DA                  8    0.272  38.611  -0.642   0.257   0.016   0.727
E D  3W2DA                  9    0.708 101.994   0.369   0.339   0.016   0.645
E E  3W2DA                 10    0.434  75.837   0.617   0.339   0.016   0.645
B L  3W2DA                 11    0.065  11.902  -0.405   0.339   0.016   0.645
E H  3W2DA                 12    0.317  57.644   0.092   0.354   0.048   0.598
E K  3W2DA                 13    0.533 109.679   0.298   0.455   0.046   0.498
E S  3W2DA                 14    0.358  41.934  -0.153   0.455   0.046   0.498
E S  3W2DA                 15    0.331  38.805  -1.354   0.354   0.048   0.598
E K  3W2DA                 16    0.490 100.711   0.156   0.455   0.046   0.498
B F  3W2DA                 17    0.104  20.893  -0.462   0.522   0.016   0.462
E T  3W2DA                 18    0.424  58.753   0.243   0.622   0.015   0.363
E G  3W2DA                 19    0.483  38.004  -1.299   0.622   0.015   0.363
B L  3W2DA                 20    0.136  24.865  -0.191   0.802   0.014   0.185
B M  3W2DA                 21    0.076  15.188  -0.930   0.802   0.014   0.185
E E  3W2DA                 22    0.307  53.633  -0.977   0.802   0.014   0.185
B N  3W2DA                 23    0.093  13.630  -0.694   0.802   0.014   0.185
B M  3W2DA                 24    0.020   4.042   0.966   0.802   0.014   0.185
B K  3W2DA                 25    0.229  47.208   0.239   0.802   0.014   0.185
E V  3W2DA                 26    0.311  47.770  -0.545   0.802   0.014   0.185
B L  3W2DA                 27    0.089  16.223   0.017   0.660   0.049   0.291
B Y  3W2DA                 28    0.087  18.699  -0.513   0.455   0.046   0.498
E D  3W2DA                 29    0.418  60.176  -0.728   0.181   0.016   0.803
E D  3W2DA                 30    0.456  65.753  -0.145   0.113   0.043   0.844
E N  3W2DA                 31    0.561  82.204  -0.505   0.113   0.043   0.844
B H  3W2DA                 32    0.266  48.367  -0.033   0.064   0.216   0.721
B V  3W2DA                 33    0.141  21.626  -0.047   0.079   0.592   0.329
E S  3W2DA                 34    0.542  63.546   0.790   0.023   0.655   0.322
E A  3W2DA                 35    0.343  37.788  -0.444   0.022   0.552   0.426
E I  3W2DA                 36    0.555 102.601   0.485   0.022   0.552   0.426
E N  3W2DA                 37    0.635  93.023  -0.553   0.022   0.359   0.619
B V  3W2DA                 38    0.178  27.328   0.553   0.021   0.451   0.528
E K  3W2DA                 39    0.486 100.052  -0.022   0.022   0.552   0.426
E S  3W2DA                 40    0.332  38.875  -1.515   0.022   0.359   0.619
E I  3W2DA                 41    0.572 105.876  -1.808   0.056   0.142   0.802
E D  3W2DA                 42    0.577  83.117  -1.663   0.052   0.084   0.864
E Q  3W2DA                 43    0.431  77.030   0.431   0.064   0.216   0.721
B F  3W2DA                 44    0.205  41.123  -0.459   0.022   0.359   0.619
B L  3W2DA                 45    0.142  26.000  -0.470   0.022   0.359   0.619
E Y  3W2DA                 46    0.303  64.772  -0.486   0.022   0.359   0.619
B F  3W2DA                 47    0.116  23.301  -0.020   0.023   0.655   0.322
B D  3W2DA                 48    0.112  16.139  -0.181   0.018   0.846   0.136
B L  3W2DA                 49    0.023   4.193   0.902   0.011   0.918   0.071
B I  3W2DA                 50    0.071  13.209  -0.261   0.011   0.918   0.071
B Y  3W2DA                 51    0.050  10.770   0.944   0.018   0.846   0.136
B S  3W2DA                 52    0.232  27.237   0.306   0.022   0.552   0.426
B I  3W2DA                 53    0.160  29.618  -1.106   0.021   0.451   0.528
E K  3W2DA                 54    0.494 101.719  -0.925   0.022   0.552   0.426
E D  3W2DA                 55    0.476  68.534  -1.525   0.022   0.359   0.619
B T  3W2DA                 56    0.253  35.105  -0.763   0.021   0.279   0.699
E K  3W2DA                 57    0.574 118.175  -0.393   0.021   0.279   0.699
E L  3W2DA                 58    0.318  58.226  -0.800   0.021   0.279   0.699
E G  3W2DA                 59    0.447  35.203  -0.628   0.021   0.279   0.699
E N  3W2DA                 60    0.566  82.848  -1.172   0.021   0.279   0.699
B Y  3W2DA                 61    0.200  42.655  -0.558   0.022   0.359   0.619
E D  3W2DA                 62    0.441  63.534  -1.293   0.021   0.279   0.699
E N  3W2DA                 63    0.283  41.402   0.472   0.022   0.552   0.426
B V  3W2DA                 64    0.039   6.056   0.137   0.021   0.756   0.223
B R  3W2DA                 65    0.171  39.113   0.860   0.018   0.846   0.136
B V  3W2DA                 66    0.076  11.620  -0.304   0.018   0.846   0.136
E E  3W2DA                 67    0.253  44.199  -0.277   0.021   0.756   0.223
B F  3W2DA                 68    0.057  11.380  -0.121   0.004   0.514   0.481
E K  3W2DA                 69    0.392  80.593   0.302   0.005   0.262   0.733
E N  3W2DA                 70    0.256  37.552  -0.462   0.118   0.150   0.732
E K  3W2DA                 71    0.475  97.666   0.213   0.802   0.014   0.185
E D  3W2DA                 72    0.504  72.670  -0.185   0.879   0.010   0.111
B L  3W2DA                 73    0.135  24.719   0.286   0.802   0.014   0.185
B A  3W2DA                 74    0.076   8.408  -1.424   0.802   0.014   0.185
E D  3W2DA                 75    0.532  76.632   0.696   0.717   0.014   0.269
E K  3W2DA                 76    0.432  88.945   0.811   0.717   0.014   0.269
B Y  3W2DA                 77    0.087  18.656   0.111   0.522   0.016   0.462
E K  3W2DA                 78    0.464  95.486   0.722   0.257   0.016   0.727
E D  3W2DA                 79    0.699 100.784  -0.977   0.058   0.017   0.925
E K  3W2DA                 80    0.529 108.836   0.858   0.018   0.047   0.935
E Y  3W2DA                 81    0.437  93.323   1.071   0.021   0.279   0.699
B V  3W2DA                 82    0.033   5.041   0.715   0.022   0.552   0.426
B D  3W2DA                 83    0.093  13.430   0.342   0.023   0.655   0.322
B V  3W2DA                 84    0.045   6.993   0.008   0.018   0.846   0.136
B F  3W2DA                 85    0.062  12.423   0.390   0.021   0.756   0.223
B G  3W2DA                 86    0.040   3.187  -1.128   0.023   0.655   0.322
B A  3W2DA                 87    0.040   4.397  -0.042   0.023   0.655   0.322
B N  3W2DA                 88    0.175  25.576  -0.581   0.022   0.552   0.426
B Y  3W2DA                 89    0.135  28.764  -0.624   0.022   0.359   0.619
B Y  3W2DA                 90    0.211  45.133  -1.605   0.021   0.279   0.699
B Y  3W2DA                 91    0.179  38.316  -1.890   0.022   0.359   0.619
E Q  3W2DA                 92    0.323  57.741  -0.456   0.023   0.655   0.322
B C  3W2DA                 93    0.035   4.942  -0.855   0.021   0.756   0.223
B Y  3W2DA                 94    0.270  57.614  -0.552   0.018   0.846   0.136
B F  3W2DA                 95    0.144  28.841  -0.409   0.023   0.655   0.322
E S  3W2DA                 96    0.319  37.375  -0.918   0.022   0.359   0.619
E K  3W2DA                 97    0.556 114.472  -2.070   0.019   0.141   0.840
E K  3W2DA                 98    0.575 118.277  -1.041   0.064   0.216   0.721
E T  3W2DA                 99    0.426  59.031  -1.176   0.066   0.296   0.638
E N  3W2DA                100    0.346  50.640  -1.560   0.069   0.386   0.545
E D  3W2DA                101    0.329  47.409  -1.639   0.069   0.386   0.545
B I  3W2DA                102    0.266  49.173  -1.199   0.135   0.317   0.548
E N  3W2DA                103    0.346  50.625  -1.603   0.125   0.227   0.648
E S  3W2DA                104    0.446  52.306  -1.518   0.113   0.087   0.800
E H  3W2DA                105    0.535  97.407  -2.455   0.052   0.084   0.864
E Q  3W2DA                106    0.452  80.799  -1.417   0.056   0.142   0.802
E T  3W2DA                107    0.460  63.816  -1.251   0.020   0.205   0.775
E D  3W2DA                108    0.485  69.845  -1.170   0.022   0.359   0.619
E K  3W2DA                109    0.486  99.888  -1.040   0.021   0.279   0.699
E R  3W2DA                110    0.433  99.180  -0.399   0.020   0.205   0.775
E K  3W2DA                111    0.287  59.139  -0.167   0.005   0.336   0.660
B T  3W2DA                112    0.167  23.135   0.390   0.004   0.616   0.381
B C  3W2DA                113    0.041   5.813   0.147   0.002   0.816   0.182
B M  3W2DA                114    0.046   9.265  -0.650   0.002   0.816   0.182
B Y  3W2DA                115    0.063  13.506  -0.611   0.003   0.718   0.279
B G  3W2DA                116    0.056   4.391  -2.115   0.021   0.451   0.528
B G  3W2DA                117    0.065   5.139  -1.614   0.021   0.451   0.528
B V  3W2DA                118    0.044   6.763  -0.164   0.023   0.655   0.322
B T  3W2DA                119    0.118  16.408   0.689   0.022   0.552   0.426
E E  3W2DA                120    0.300  52.393   0.162   0.022   0.359   0.619
B H  3W2DA                121    0.269  48.913  -0.334   0.019   0.141   0.840
E N  3W2DA                122    0.586  85.746  -0.965   0.052   0.084   0.864
E G  3W2DA                123    0.528  41.577  -1.520   0.052   0.084   0.864
E N  3W2DA                124    0.453  66.348  -1.163   0.056   0.142   0.802
E Q  3W2DA                125    0.511  91.175  -0.128   0.064   0.216   0.721
B L  3W2DA                126    0.228  41.747  -0.533   0.056   0.142   0.802
E D  3W2DA                127    0.603  86.863  -1.482   0.052   0.084   0.864
E K  3W2DA                128    0.553 113.732  -0.301   0.052   0.084   0.864
E Y  3W2DA                129    0.514 109.735  -1.173   0.019   0.141   0.840
E R  3W2DA                130    0.282  64.601   0.338   0.005   0.336   0.660
E S  3W2DA                131    0.289  33.906   0.406   0.003   0.718   0.279
B I  3W2DA                132    0.023   4.292   1.014   0.002   0.816   0.182
B T  3W2DA                133    0.070   9.723  -0.485   0.001   0.900   0.099
B V  3W2DA                134    0.017   2.551   1.115   0.001   0.900   0.099
B R  3W2DA                135    0.119  27.320   0.014   0.001   0.900   0.099
B V  3W2DA                136    0.020   3.135   1.108   0.001   0.900   0.099
B F  3W2DA                137    0.097  19.488   0.215   0.002   0.816   0.182
B E  3W2DA                138    0.194  33.839   0.092   0.003   0.718   0.279
E D  3W2DA                139    0.483  69.600  -0.222   0.004   0.138   0.858
E G  3W2DA                140    0.532  41.900  -1.571   0.018   0.047   0.935
E K  3W2DA                141    0.515 105.853  -0.100   0.005   0.336   0.660
E N  3W2DA                142    0.390  57.037  -0.103   0.004   0.616   0.381
E L  3W2DA                143    0.400  73.313  -0.056   0.004   0.616   0.381
E L  3W2DA                144    0.334  61.247   0.020   0.004   0.514   0.481
E S  3W2DA                145    0.416  48.732  -1.018   0.004   0.514   0.481
B F  3W2DA                146    0.218  43.692  -0.500   0.023   0.655   0.322
E D  3W2DA                147    0.391  56.343   0.644   0.018   0.846   0.136
B V  3W2DA                148    0.049   7.593   0.480   0.011   0.918   0.071
E Q  3W2DA                149    0.391  69.761   0.565   0.018   0.846   0.136
B T  3W2DA                150    0.103  14.314  -0.302   0.023   0.655   0.322
E N  3W2DA                151    0.591  86.581  -0.080   0.020   0.205   0.775
E K  3W2DA                152    0.428  88.101   0.416   0.018   0.047   0.935
E K  3W2DA                153    0.516 106.182  -0.202   0.019   0.141   0.840
E K  3W2DA                154    0.459  94.313   0.904   0.021   0.451   0.528
B V  3W2DA                155    0.061   9.453   0.526   0.087   0.683   0.230
B T  3W2DA                156    0.148  20.528   0.808   0.087   0.683   0.230
B A  3W2DA                157    0.058   6.381   0.199   0.273   0.587   0.140
B Q  3W2DA                158    0.207  36.899   0.668   0.391   0.466   0.143
B E  3W2DA                159    0.158  27.568   0.161   0.594   0.285   0.121
B L  3W2DA                160    0.050   9.137   0.799   0.701   0.107   0.192
B D  3W2DA                161    0.065   9.338  -0.153   0.831   0.044   0.125
B Y  3W2DA                162    0.111  23.614   0.937   0.938   0.007   0.055
E L  3W2DA                163    0.274  50.133   1.270   0.938   0.007   0.055
B T  3W2DA                164    0.028   3.939   0.484   0.975   0.003   0.022
B R  3W2DA                165    0.127  28.969   0.770   0.975   0.003   0.022
E H  3W2DA                166    0.351  63.883   1.179   0.975   0.003   0.022
B Y  3W2DA                167    0.212  45.240   1.647   0.975   0.003   0.022
B L  3W2DA                168    0.024   4.431   0.407   0.938   0.007   0.055
B V  3W2DA                169    0.227  34.951   0.704   0.938   0.007   0.055
E K  3W2DA                170    0.658 135.371   0.382   0.802   0.014   0.185
E N  3W2DA                171    0.437  64.035   0.660   0.522   0.016   0.462
B K  3W2DA                172    0.170  35.051   0.863   0.354   0.048   0.598
E K  3W2DA                173    0.333  68.580   0.134   0.216   0.235   0.548
B L  3W2DA                174    0.064  11.663  -0.177   0.069   0.386   0.545
B Y  3W2DA                175    0.155  33.123  -0.162   0.069   0.386   0.545
E E  3W2DA                176    0.339  59.153  -0.398   0.066   0.296   0.638
E F  3W2DA                177    0.349  70.084  -0.641   0.018   0.088   0.893
E N  3W2DA                178    0.709 103.842  -1.279   0.018   0.019   0.964
E N  3W2DA                179    0.660  96.580  -2.042   0.018   0.019   0.964
E S  3W2DA                180    0.320  37.516  -1.843   0.018   0.019   0.964
E P  3W2DA                181    0.514  72.951   0.658   0.019   0.141   0.840
B Y  3W2DA                182    0.118  25.281   0.136   0.004   0.420   0.576
E E  3W2DA                183    0.414  72.291   0.046   0.004   0.616   0.381
E T  3W2DA                184    0.432  59.960   0.825   0.003   0.718   0.279
B G  3W2DA                185    0.066   5.163  -0.800   0.002   0.816   0.182
B Y  3W2DA                186    0.195  41.629   1.127   0.001   0.959   0.040
B I  3W2DA                187    0.035   6.512   0.179   0.000   0.983   0.017
B K  3W2DA                188    0.185  38.013   1.176   0.000   0.983   0.017
B F  3W2DA                189    0.032   6.342   0.188   0.001   0.959   0.040
B I  3W2DA                190    0.116  21.386   0.601   0.001   0.900   0.099
E E  3W2DA                191    0.312  54.576   0.097   0.004   0.616   0.381
E N  3W2DA                192    0.492  72.029   0.138   0.004   0.085   0.910
E E  3W2DA                193    0.715 124.858  -1.010   0.003   0.003   0.994
E N  3W2DA                194    0.736 107.677  -0.032   0.005   0.015   0.979
E S  3W2DA                195    0.403  47.255   1.229   0.004   0.514   0.481
B F  3W2DA                196    0.153  30.807   1.027   0.001   0.900   0.099
B W  3W2DA                197    0.202  48.533   0.742   0.001   0.959   0.040
B Y  3W2DA                198    0.048  10.322   0.925   0.001   0.900   0.099
B D  3W2DA                199    0.069   9.957  -1.423   0.018   0.846   0.136
B M  3W2DA                200    0.034   6.903  -0.118   0.022   0.552   0.426
B M  3W2DA                201    0.054  10.865   0.430   0.004   0.514   0.481
B P  3W2DA                202    0.189  26.862  -0.339   0.004   0.420   0.576
B A  3W2DA                203    0.206  22.679   0.368   0.019   0.141   0.840
E P  3W2DA                204    0.604  85.693  -0.747   0.018   0.019   0.964
E G  3W2DA                205    0.592  46.590  -2.750   0.016   0.005   0.979
E D  3W2DA                206    0.589  84.933  -1.852   0.018   0.019   0.964
E K  3W2DA                207    0.363  74.731  -1.202   0.058   0.017   0.925
B F  3W2DA                208    0.218  43.833  -1.861   0.053   0.043   0.903
E D  3W2DA                209    0.357  51.415  -0.472   0.115   0.016   0.868
E Q  3W2DA                210    0.293  52.312  -0.504   0.455   0.046   0.498
E S  3W2DA                211    0.436  51.123  -0.557   0.538   0.173   0.289
E K  3W2DA                212    0.373  76.808   0.290   0.502   0.102   0.396
B Y  3W2DA                213    0.077  16.455   0.474   0.538   0.173   0.289
B L  3W2DA                214    0.084  15.472   0.145   0.578   0.229   0.194
E M  3W2DA                215    0.341  68.254   0.895   0.478   0.309   0.214
B M  3W2DA                216    0.161  32.236   0.250   0.478   0.309   0.214
B Y  3W2DA                217    0.121  25.879   0.449   0.321   0.252   0.427
E N  3W2DA                218    0.559  81.867   0.058   0.199   0.152   0.649
E D  3W2DA                219    0.528  76.085  -0.395   0.053   0.043   0.903
E N  3W2DA                220    0.340  49.732  -1.013   0.053   0.043   0.903
E K  3W2DA                221    0.453  93.162   0.353   0.020   0.205   0.775
E M  3W2DA                222    0.361  72.296   0.566   0.022   0.552   0.426
B V  3W2DA                223    0.092  14.110   0.888   0.022   0.552   0.426
E D  3W2DA                224    0.476  68.635   0.651   0.022   0.359   0.619
B S  3W2DA                225    0.201  23.557  -0.823   0.020   0.205   0.775
E K  3W2DA                226    0.632 130.105  -0.167   0.021   0.279   0.699
E D  3W2DA                227    0.443  63.851   1.064   0.005   0.336   0.660
B V  3W2DA                228    0.081  12.404   0.615   0.004   0.616   0.381
B K  3W2DA                229    0.182  37.479   0.321   0.002   0.816   0.182
B I  3W2DA                230    0.046   8.584   0.422   0.001   0.900   0.099
B E  3W2DA                231    0.080  13.993  -0.135   0.001   0.959   0.040
B V  3W2DA                232    0.023   3.612   1.042   0.001   0.959   0.040
B Y  3W2DA                233    0.114  24.362   0.811   0.001   0.959   0.040
B L  3W2DA                234    0.032   5.786   0.438   0.001   0.959   0.040
B T  3W2DA                235    0.129  17.865  -0.058   0.001   0.959   0.040
E T  3W2DA                236    0.224  31.055   0.128   0.001   0.900   0.099
E K  3W2DA                237    0.360  74.134  -0.134   0.004   0.514   0.481
E K  3W2DA                238    0.630 129.570  -0.610   0.020   0.205   0.775
E K  3W2DA                239    0.835 171.759   0.688   0.003   0.003   0.994

Explain the output.  Go back.
```

---
